# Hemostatic Patches Based on Crosslinked Chitosan Films Applied in Interventional Procedures

**DOI:** 10.3390/polym13152402

**Published:** 2021-07-22

**Authors:** Moon Hyun Lee, Dae Ryeong Lee, Joon Woo Chon, Dong June Chung

**Affiliations:** Department of Polymer Science and Engineering, Sungkyunkwan University, Suwon 16419, Korea; bluehyun93@skku.edu (M.H.L.); migiwoju07@skku.edu (D.R.L.); chonjoon@skku.edu (J.W.C.)

**Keywords:** crosslinked chitosan film, EDC/NHS coupling reaction, genipin, glutaraldehyde, hemostasis, thrombin coating

## Abstract

In this study, we manufactured biocompatible hemostatic crosslinked chitosan (CS) patches and analyzed their physicochemical and biological properties for femoral arterial puncture applications. CS is a representative hemostatic material but has some drawbacks, such as swelling, shrinkage, and brittleness. Thus, it was crosslinked via a 1-ethyl-3-(3-dimethyl aminopropyl) carbodiimide (EDC)/*N*-hydroxysuccinimide (NHS) coupling reaction and a nucleophilic addition reaction with citric acid (CA), glutaraldehyde (GTA), and genipin (GP) to remedy its shortcomings. The CSCA (crosslinked CS with CA/EDC), CSGTA (crosslinked CS with GTA), and CSG (crosslinked CS with GP) films showed low swelling degrees and good mechanical properties (excluding CSCA) compared with those of neat CS films. Additionally, every crosslinked CS film coated with thrombin (TB-CS) showed enhanced hemostatic ability in the whole blood clotting and activated partial thromboplastin time tests. Furthermore, the CSCA, CSGTA, and CSGP were nontoxic in an in vitro cell cytotoxicity test (3-(4,5-dimethylthiazolyl-2)-2,5-diphenyltetrazolium bromide assay) using L-929 mouse fibroblasts cells.

## 1. Introduction

Femoral arterial punctures that occur after stent insertion surgeries trigger uncontrolled bleeding. Therefore, hemostasis at the arterial puncture area is essential. Arterial hemostasis methods are performed traditionally by manual compression over the wound site or using compression devices, such as bioresorbable plugs and sutures. These methods can induce peripheral blood vessel complications and cause more pain for patients because of the hemostasis time extension [1,2]. Additionally, patches and pads, such as the Syvek patch (poly-*N*-acetyl glucosamine), the Clo-Sur pad, and the Chito-Seal pad (based on chitosan), have been studied covering the surface of the function area after catheter removal [3,4]. However, using non-adhesive hemostatic pads may cause infection and cell necrosis. Furthermore, the wound site may not be sealed completely. Thus, a thrombin-coated chitosan (CS) hemostatic patch was developed to resolve these limitations and enhance the hemostatic effect by adding a hydrocolloid adhesive patch to compress and seal the punctured area for stent insertion.

CS is a biocompatible natural polymer and a representative hemostatic material forming cationic clusters that interact with the anions of red blood cells. However, applying CS in hemostatic patches is difficult because of its brittleness and swelling property under moisture conditions [5,6,7]. For these reasons, CS was crosslinked with various crosslinkers, such as citric acid (CA), glutaraldehyde (GTA), and genipin (GP), to resolve the above-mentioned shortcomings.

The representative methods of crosslinking CS are 1-ethyl-3-(3-dimethyl aminopropyl) carbodiimide (EDC)/*N*-hydroxysuccinimide (NHS) coupling reactions and nucleophilic addition reactions [8,9,10,11,12]. CA is a biocompatible and inexpensive organic acid with tricarboxylic acid groups [11,12]. GTA is a widely used crosslinker that enhances water resistance and mechanical properties despite its cytotoxicity [13,14,15,16]. GP extracted from the Gardenia jasminoides fruits is a biocompatible iridoid compound with a cylclopentanopyran structure. It is used in herbal medicine, dark blue pigments, drug delivery, and hydrogels because of its low cytotoxicity and anti-inflammatory properties [17,18,19].

In this study, the three previously mentioned kinds of crosslinked CS films were synthesized via EDC/NHS coupling and nucleophilic addition reactions to improve CS’s swelling and mechanical properties. Furthermore, these were coated with thrombin to improve their blood clotting effect. Hemostatic crosslinked CS patches were investigated to confirm their physicochemical and biological properties using Fourier transform infrared spectroscopy (FT-IR), a solubility test, swelling and tensile tests, surface elemental analysis, a whole blood test, an activated partial thromboplastin time (aPTT) test, and 3-(4,5-dimethylthiazolyl-2)-2,5-diphenyltetrazolium bromide (MTT) assay.

## 2. Experimental Section

### 2.1. Materials

CS (Mw: 190–370 kDa, degree of deacetylation: ≥75%), thrombin (from bovine plasma, ≥60 NIH units/mg protein), phosphate-buffered saline (PBS, pH 7.4), kaolin, calcium chloride (CaCl_2_, purity: ≥93.0%), L-α-phosphatidylethanolamine (cephalin) from egg yolk (purity: ≥97.0%), penicillin–streptomycin, Dulbecco’s Modified Eagle’s Medium (DMEM), fetal bovine serum (FBS), and MTT formazan (purity: ≥97.0%) were purchased from Sigma-Aldrich Chem. Co. (St. Louis, MO, USA). CA (citric acid, purity: >98.0%), 1-ethyl-3-(3-dimethylaminopropyl) carbodiimide hydrochloride (EDC, purity: >98.0%), *N*-hydroxysuccinimide (NHS, purity: >98.0%), and potassium bromide (KBr, purity: >99.0%) were purchased from Tokyo Chemical Industry Co., Ltd. (Tokyo, Japan). GTA (2.6 M, diluted in water, purity: ≥98.0%) was purchased from Honeywell Fluka (Charlotte, NC, USA). GP (purity: >98.0%) was purchased from Wako Co., Ltd. (Tokyo, Japan). Acetic acid (purity: >99.7%) and ethanol (purity: >99.5%) were purchased from DAEJUNG Chemical & Metal Co., Ltd. (Siheung, Korea). Sodium hydroxide (NaOH, 98%) was purchased from SAMCHUN Chemical Pure Co. (Pyeongtaek, Korea). A hydrocolloid sheet was supplied by ANYTAPE Co., Ltd. (Hwaseong, Korea). Canine whole blood for hemostatic experiments was obtained from Korea Animal Blood Bank (Sokcho, Korea). L-929 mouse fibroblast cells were purchased from Korean Cell Line Bank (Seoul, Korea) for cytotoxicity evaluation.

### 2.2. Crosslinking Methods of CS Films

A neat (uncrosslinked) CS film was prepared using the solution casting method. First, CS powder (1.5 g) was dissolved in 100 mL of 1.0% (*v*/*v*) aqueous acetic acid for 24 h at room temperature. Then, 100 mL of the obtained CS solution was poured into a 125 mm × 125 mm polystyrene square petri dish (SPL life science, Pocheon, Korea) and dried at 37 °C for 24 h. Finally, a transparent CS film was obtained and used for the control sample.

**Scheme 1:**
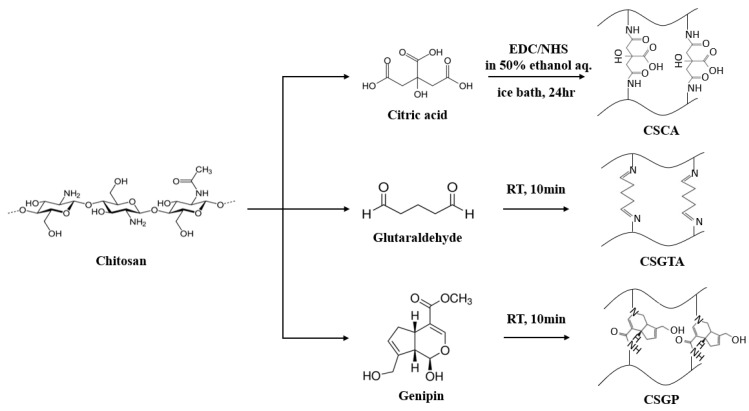
Crosslinking reaction procedure between CS and three kinds of crosslinkers (CA, GTA, and GP).

Crosslinked CS films were synthesized following the procedure in Scheme 1. First, CA, EDC, and NHS were mixed in 50.0% (*v*/*v*) ethanol and water solution at 0 °C for 90 min following their respective molar ratios in Table 1. The numbers in the sample names refer to the number of crosslinkers and coupling reagents. Then, 100 mL of 1.5 wt% CS in acetic acid aqueous solution was added to the 100 mL CA solution with EDC and NHS in an ice bath and stirred for 24 h. Finally, the obtained CSCA solution (100 mL) was poured into a 125 mm × 125 mm polystyrene square dish and dried at 37 °C for 24 h in a convection oven.

The CSGTA and CSGP films were prepared using GTA and GP, respectively, instead of EDC/NHS as the crosslinker. Predetermined amounts of GTA or GP (Table 1) were directly added to 100 mL of the 1.5 wt% CS solution. The CSGTA and CSGP mixtures were stirred using a mechanical stirrer at room temperature for 10 min and poured into 125 mm × 125 mm polystyrene square dishes. After drying, every CS film was immersed in 80% (*v*/*v*) aqueous ethanol solution with NaOH (2.0 wt%) for 2 h to remove unreacted moieties and then rinsed with distilled water three times.

### 2.3. Preparation of the TB-CS Films

TB-CS films were manufactured using an air spray gun (0.22 MPa, AIRTEX-APC-008, Osaka, Japan). Crosslinked CS films (1.0 cm × 1.0 cm) were coated with thrombin solution (dissolved in 0.01 M PBS at 9.9 mg/mL). The final solid thrombin contents on the attained TB-CS films were 320, 640, and 960 µg thrombin/cm^2^. These TB-CS films were dried overnight at 37 °C in a convection oven.

### 2.4. Analysis of Physicochemical Properties

#### 2.4.1. Confirmation of the Crosslinking Reaction of the CS Films

The FT-IR spectra (Nicolet iS5 FT-IR, Thermo Fisher Scientific, Waltham, MA, USA) of the neat CS, CSCA, CSGTA, and CSGP were measured to determine their chemical structures after crosslinking reactions. Samples of CSCA 1, CSGTA 1, and CSGP 1, having the lowest number of crosslinking agents, were used for this measurement. For FT-IR specimens, KBr and four kinds of CS films were crushed using a cryo-mill (SPEX 6770, SPEX Sample Prep, Metuchen, NJ, USA) and dried at 70 °C in a vacuum oven for 24 h.

The neat CS and crosslinked CS films were immersed in 5.0 mL of 1.0% (*v*/*v*) acetic acid aqueous solution to verify the crosslinking reaction. The dissolution behaviors of the CS films were observed for 24 h at room temperature.

#### 2.4.2. Swelling Test

Various 1.0 cm × 1.0 cm crosslinked CS films were prepared and immersed in a 5.0 mL PBS solution (pH 7.4) for 24 h to investigate the swelling behaviors of the crosslinked CS films. Then, the films were weighed after removing the remaining liquid from the films. The swelling degrees of the crosslinked CS films were calculated using the following equation, where *W_f_* is the weight of the CS films at a wet state and *W_o_* is the weight of the CS films at a dry state:

Swelling degree (%) = [(*W_f_* − *W_o_*)/*W_o_*] × 100 (1)

#### 2.4.3. Mechanical Properties

The mechanical properties of the crosslinked CS films were evaluated using a universal testing machine (UTM, ElectroPlus E3000 Linear-Torsion, Instron, Norwood, MA, USA). The specimens were prepared according to ASTM D882. The load cell and tension speed were set to 250 N and 10.0 mm/min, respectively.

### 2.5. Analysis of Biological Properties

#### 2.5.1. In Vitro Blood Coagulation Assay

The aPTT and whole blood clotting tests were performed using the slide method to evaluate the hemostatic ability of the crosslinked CS films [20,21]. We prepared 1.0 cm × 1.0 cm TB-CS film specimens and used cover glass as a control. For the aPTT test, platelet-poor plasma that was separated from the canine whole blood was mixed with kaolin (surface activator) and cephalin (clotting activation factor and a platelet substitute). Then, 50.0 µL each of the above-mentioned solutions and a 0.025M calcium chloride solution (another clotting activation factor) were dropped on the specimens, which were then stirred slowly until platelet clots appeared. aPTT was recorded using a stopwatch until the platelet clots turned up.

For the whole blood clotting test, 100 µL of canine whole blood was dropped on the crosslinked CS films, which were then stirred slowly to manifest thrombus formation. Similar to the aPTT test, blood clotting time was recorded using a stopwatch until thrombi appeared.

#### 2.5.2. In Vitro Cell Cytotoxicity Test (MTT Assay)

An MTT assay was performed using L-929 mouse fibroblasts cells according to ISO 10993-5 guidelines to confirm the cytotoxicity of each CS film. Every crosslinked CS film was consecutively washed with ethanol and PBS solution for 24 h. For the assay, MTT was dissolved in PBS solution (5.0 mg/mL). Every CS film was immersed in a DMEM medium containing 10% FBS and 1% penicillin (4.0 g/20.0 mL) for 24 h at 36.5 °C for exudation. The extracted solution was diluted to various concentrations using the DMEM medium (100%, 50%, and 25%). Fibroblast cells (NCTC clone 929; L-929, KCLB No. 10001) were seeded (1.0 × 10^4^ cells) into each well of 96-well tissue culture polystyrene dish plates (SPL Life Science, Pocheon, Korea) and cultured for 24 h at 36.5 °C and 5% CO_2_ conditions in an incubator (SL-205C, Thermo Fisher Scientific, Waltham, MA, USA). After cultivation, the incubated medium was substituted with a fresh DMEM medium (100 µL). Then, an MTT (25.0 µL) solution was added to each well. After additional incubation for 4 h, the cultured mixtures were removed. Dimethyl sulfoxide (DMSO, 100 µL) was added to each well to dissolve the synthesized formazan crystals. The ultraviolet–visible (UV–Vis) absorbance of the solutions in each well was measured at 570 nm using a Varioskan LUX multimode microplate reader (Thermo Fisher Scientific Co., Ltd., Waltham, MA, USA) to determine the cell viability.

## 3. Results and Discussions

### 3.1. Physicochemical Properties of the Crosslinked CS Films

#### 3.1.1. Confirmation of the Crosslinking Reaction of the CS Films

**Figure 1:**
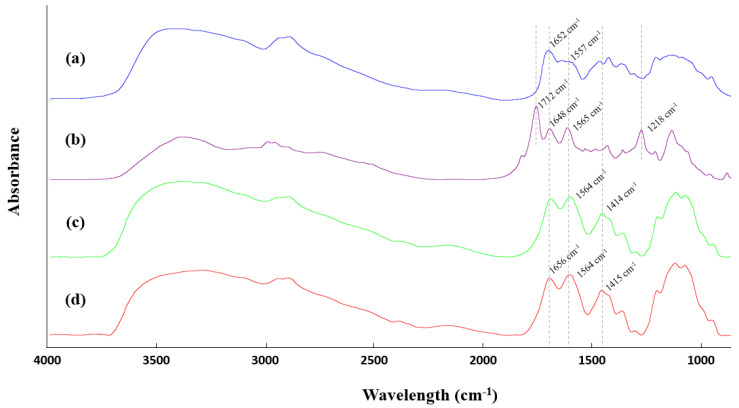
FT-IR spectra of the crosslinked CS films: (**a**) neat CS, (**b**) CSCA 1, (**c**) CSGTA 1, and (**d**) CSGP 1.

Figure 1 shows the FT-IR spectra of the neat CS and crosslinked CS films (CSCA, CSGTA, and CSGP) measured to confirm their chemical structures, which contain crosslinking bonds owing to newly formed amide and imine bonds detected between 1800 and 1200 cm^−1^. The neat CS showed amine and amide bond peaks at 1652 and 1557 cm^−1^, respectively, corresponding to C=O (stretch) and N–H (bending) in CS. The peaks of CSCA were observed at 1648, 1565, 1712, and 1218 cm^−1^, corresponding to C=O (stretch)/N–H (bending) of the amide bond and C=O (stretch)/C–O (stretch) of the carbonyl group in CA, respectively. These data show that CSCA has new amide bonds formed through the EDC/NHS coupling reaction between carboxylic acid in CA and primary amine in CS [12].

In the case of CSGTA, infrared (IR) peaks were observed at 1564 and 1414 cm^−1^, corresponding to C=N (stretch) of the imine bond and C–H (bending) between the aldehyde group of GTA and primary amine in CS due to a nucleophilic addition reaction [14,22]. Likewise, the CSGP showed peaks at 1656, 1564, and 1415 cm^−1^, corresponding to C=O (stretch)/N–H (bending) of the amide bond and C–H (bending) of the methyl ester group in GP, respectively [19,23]. However, using only FT-IR spectra to confirm the crosslinking reaction of CSCA, CSGTA, and CSGP films was difficult and insufficient because their IR peaks overlapped with those of the primary amine and newly formed amide bonding of neat and crosslinked CS. Thus, a solubility test was performed to determine whether the CS films were crosslinked or not.

**Figure 2:**
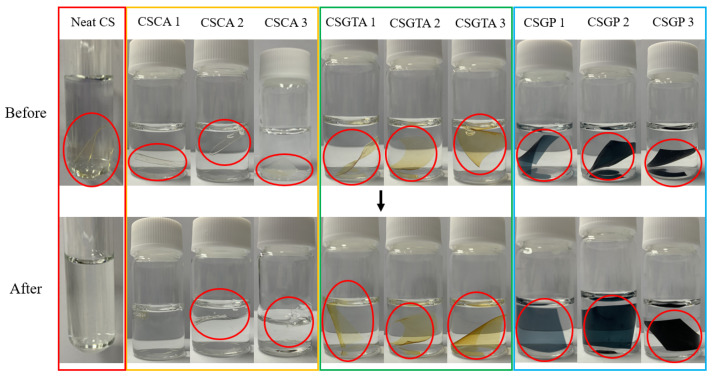
Solubility behavior images of the crosslinked CS films with various crosslinker concentrations under an acidic condition (pH 2.6). Undissolved CS films in aqueous 1.0% acetic acid solution (pH 2.6) immersed for 24 h were marked by red circles.

Uncrosslinked CS is soluble in acidic aqueous solutions because primary amine groups of the CS are converted to positive ions (–NH_3_^+^) through protonation. Thus, it was assumed that uncrosslinked CS would be soluble under acidic conditions. However, if the CS is crosslinked, the mobility and repulsion of the CS chains decreased, and solvent molecules hardly penetrated the CS molecular chains [22,24]. These mainly influenced the solubility decrease in crosslinked CS. Figure 2 and Table 2 show the solubility test results of CS films under an acidic condition (pH 2.6). The neat CS film completely dissolved in the acidic aqueous solution, but the CSGTA and CSGP films were insoluble even at a low pH. These results mean that the protonation of amine groups significantly affects the solubility of the neat CS and the crosslinked CS films [24].

Similarly, the CSCA films were insoluble under acidic conditions in the case of high crosslinker concentrations. It is supposed that the crosslinking reaction of the CSCA 2 and CSCA 3 films proceeded successfully. However, CSCA 1 dissolved in an acidic environment, although an amide bond was detected in its structure on the basis of the FT-IR spectrum. This result infers that it was partially crosslinked and was easily penetrated by solvent molecules because of its low crosslinking density compared with those of CSCA 2 and CSCA 3.

#### 3.1.2. Swelling Behavior of Crosslinked CS Films in Neutral Conditions

**Figure 3:**
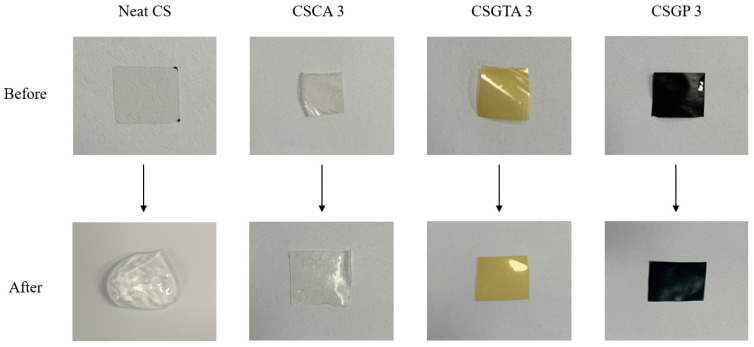
Morphology changes of the crosslinked CS films before and after the swelling test in PBS (pH 7.4) for 24 h.

The swelling behaviors of the crosslinked CS films were investigated using a PBS solution (pH 7.4) for 24 h. The results were summarized with respect to solubility in Table 2. As shown in Table 2, the swelling degrees of the crosslinked CS films were less than those of the neat CS film. The neat CS film showed a very high swelling degree (almost 1000%) and a crushed shape (Figure 3) after the swelling test because CS is insoluble under neutral conditions (but is soluble in a 1.0% acetic acid solution, pH 2.6). However, it still has high water absorption by original hydrophilic groups (such as hydroxyl and amine bonds) [25,26]. Conversely, the crosslinked CS films showed lower swelling degrees and maintained almost their original shapes (except CSCA 1) after the swelling test (Figure 3).

The CSCA films showed different swelling degrees depending on the crosslinker concentration (EDC/NHS). Particularly, CSCA 1 showed a reduced swelling degree compared with that of the neat CS. This means that CSCA is insoluble under a neutral pH but is soluble in a 1.0% acetic acid solution (pH 2.6). Generally, the swelling degree decreases with increasing crosslinker concentration. However, the swelling behavior of the CSCA showed a different tendency. This phenomenon is related to the insufficient crosslinking reaction efficacy in the case of CSCA manufacturing, especially for CSCA 1, which was only partially crosslinked with a weak network structure. It was also inferred that the swelling degree increased with increasing amounts of residual hydrophilic groups in CS, such as carboxyl and hydroxyl groups, owing to the partially crosslinked CSCA [12].

The swelling degrees of the CSGTA and CSGP films were about five to seven times less than that of the neat CS film. This shows that the swelling degree depends on the crosslinker concentration. The decreased swelling degree was related to crosslinking density, and the crosslinking reaction promotes coherent behaviors in CS chains. Thus, stable covalent bonds were newly formed between the crosslinker (GTA and GP) and CS chains. In the case of CSGTA, the aldehyde group of GTA and primary amine in CS react to form a Shiff base. In CSGP, a secondary amide was formed by the reaction between carboxymethyl groups in GP and primary amines in CS. If these reactions are promoted, the mobility of the CS interchain is reduced. Therefore, the water resistance of the CSGTA and CSGP films was enhanced through crosslinking reactions [16,18].

#### 3.1.3. Mechanical Properties of the Crosslinked CS Films

UTM tests were performed before and after crosslinking reactions to compare the samples’ mechanical properties, such as tensile strength, elastic modulus, and elongation at break. As shown in Table 3, the neat CS film showed a tensile strength of 35.5 MPa (±1.1), an elastic modulus of 34.5 MPa (±1.1), and an elongation of 1.7% (±0.2). Conversely, the CSCA film showed a lower tensile strength and elastic modulus, but its elongation at break increased. As mentioned above, this is due to the weak interactions of the polymer chains of CSCA owing to the crosslinker’s low reactivity to CS. Additionally, unreacted crosslinker CA molecules remained and were distributed in the CS polymer chain in the CSCA films. These manifested through the weak chain interaction and the plasticizer effect [25,26].

The mechanical properties of the CSGTA and CSGP films had values higher than those of the neat CS film (almost two times higher), which tended to decrease with increasing crosslinker concentrations. Although the interaction between the CS chains becomes strong because of the good crosslinking reaction efficiency with increased crosslinker concentrations, the films exhibited brittle properties. These results mean that the mobility of the CS interchain in the CSGTA and CSGP films was more restricted by the crosslinking reactions [16,19].

### 3.2. Biological Properties of the CS Films

#### 3.2.1. In Vitro Blood Coagulation Assay

**Figure 4:**
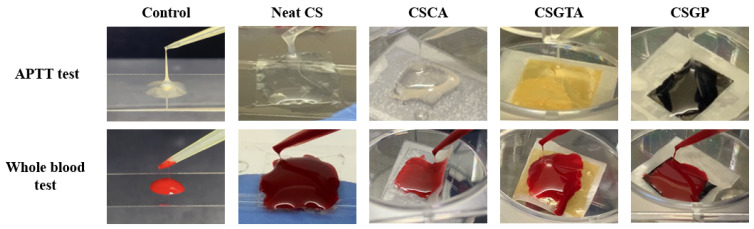
Clot-forming behaviors of the TB-CS films with canine whole blood.

In vitro blood coagulation assay was performed using the aPTT and whole blood clotting tests. These tests were conducted to confirm the hemostatic behavior of the crosslinked CS and TB-CS films. The amount of thrombin on the TB-CS films after the coating process was calculated as maximally 640 µg considering an end-on type single-layer coating of thrombin based on its molecular size (45 Å × 45 Å × 50 Å) [27]. Hemostatic behaviors can be determined through the formation of platelet clots and fibrin fibers on the TB-CS films by blood coagulation factors involved in whole blood. Figure 4 shows the formation of platelet and fibrin clots in the in vitro blood coagulation test.

The aPTT test is a representative blood anti-coagulation verification method. However, in this study, it was adapted as a hemostatic tool to confirm blood coagulation. Generally, various blood factors (factors XII, XI, X, VIII, and IX) convert prothrombin to thrombin, and thrombin makes fibrin fibers from fibrinogen. Kaolin (coagulation activator), cephalin (platelet substitute), and calcium ion (coagulation factor) in the aPTT reagents activate these steps, including the conversion of prothrombin to thrombin [28]. Thus, the aPTT results of the different samples are constant in the case of the nonexistence of external factors (blood cells and platelets) [21]. Figure 4 shows the platelet clots and fibrin fibers formed on glass and TB-CS films in the aPTT and whole blood clotting tests. These results are summarized in Table 4.

The control value demonstrates that the results of this study are very close to those of similar studies [29]. Depending on the sampling and handling of blood samples, the analysis environment, and the conditions, the results of the blood clotting time analysis may vary slightly [30].

Although CS has relatively good hemostatic properties, the CS films (including the crosslinked CS samples) without thrombin showed almost the same aPTT results within 64–69 s compared with the control (70 ± 0.3 s). This result indicates that the blood coagulation cascade did not occur using CS cations without the aid of blood cells and platelets, which were eliminated in the preparation of the aPTT solution. Thus, whole blood clotting tests were performed after thrombin coating of the crosslinked CS films (TB-CS films) to improve and confirm the hemostatic ability of the crosslinked CS films.

Cover glass as the control was observed after 280 s (±20.3). The neat CS and crosslinked CS films without coated thrombin showed shortened blood clotting times (about 100 and 90 s) compared with those of the control. From these results, non-thrombin-coated CS can make thrombi by aggregation and adherence of blood coagulation factors because of the positive charges in its molecular chain [5,6,7]. CS (a linear polysaccharide) easily forms network structures that provide a suitable environment for red blood cells and platelets to aggregate and finally adhere to the CS chain. Therefore, its structure promotes the interaction of blood components with CS and the formation of blood clots. In addition, the positive charge in the CS’s molecular chain promotes blood coagulation because it affects the activation and acceleration of platelet adhesion and aggregation. Thus, because of the network structure and positive charge in their chains, the crosslinked CS films (CSCA, CSGTA, and CSGP) enhanced the interaction of blood coagulation factors and encouraged good blood clotting behaviors [31].

The whole blood clotting times of the TB-CS films were shortened with increasing amounts of coated thrombin. However, when the amount of coated thrombin was over 640 µg/cm^2^ (the theoretically maximum amount based on an end-on type single layer), the hemostatic effect did not change drastically. For the 320 µg/cm^2^ coating samples, the clotting time was longer than those for the 640 and 960 µg/cm^2^ samples because of the insufficient amount of thrombin molecules for end-on type single-layer coating. Such tendencies were revealed in all the CSCA, CSGTA, and CSGP films.

#### 3.2.2. In Vitro Cell Cytotoxicity Test (MTT Assay)

**Figure 5:**
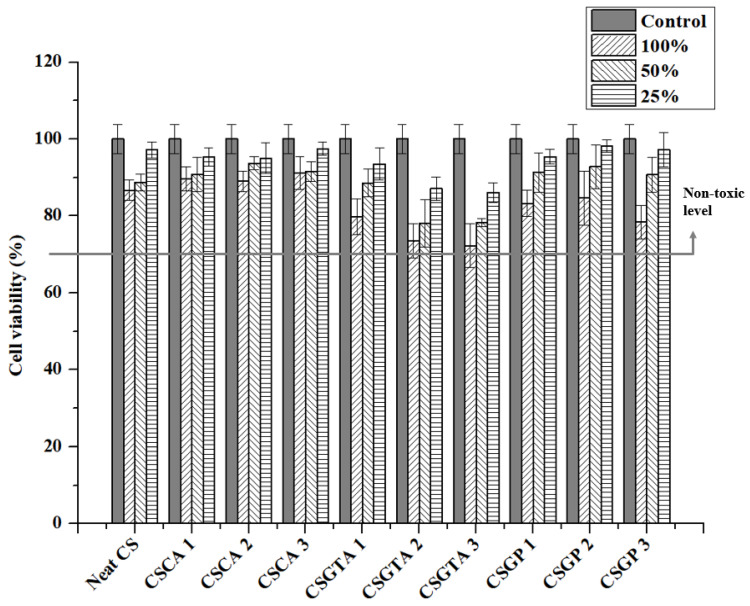
Cell viability results of the crosslinked CS films with various crosslinker concentrations from the MTT assay.

The cytotoxicity of the crosslinked CS films was investigated using an MTT assay. MTT formazan was formed by the reduction reaction of mitochondrial dehydrogenase in living cells. The absorbance of the formed formazan in a DMSO solution at 570 nm was proportional to that of the reduced MTT formazan. Thus, the amounts of living cells and cell viability were deeply related to absorbance changes [32]. Every crosslinked CS film was immersed in DMEM for 1 day, and the extracted solution was diluted using the same DMEM to 100% from a 25% concentration. Figure 5 shows the cytotoxicity test results of the crosslinked CS samples, among other biocompatibility factors. Even if the concentration of the extracts increased from 25 to 100% of the total volume of the medium, the cell viability of every crosslinked CS sample was at nontoxic levels (above 70%) based on ISO 10993-5 standards. Particularly, although the water-soluble GTA is toxic [12,13,14,15], the CSGTA film had good cell viability after washing. Therefore, the crosslinked CS films (CSCA, CSGTA, and CSGP) were biocompatible and thus may be applied in hemostatic patches.

## 4. Conclusions

We studied the physicochemical properties and biological behaviors of hemostatic crosslinked CS films. Every crosslinked CS film showed enhanced water resistance and mechanical properties compared with those of the neat CS. However, the CSCA film showed physicochemical properties inferior to those of the other crosslinked CS films (CSGTA and CSGP). In addition, the hemostatic-linked CS films coated with thrombin exhibited quick blood clotting and good aPTT results compared with those of the neat CS. Furthermore, the CSGTA film exhibited moderate cytotoxicity, but the CSCA and CSGP films were nontoxic based on ISO standards, even though their extract concentrations were up to 100%. On the basis of these results, the CSCA and CSGP films are suitable for hemostatic patches used in interventional procedures.

## Figures and Tables

**Table 1:** The molar ratios of the reactants used for the crosslinking reactions.

Sample	Reactant Mole Ratio					
	Chitosan	Citric Acid	EDC	NHS	Glutaraldehyde	Genipin
Neat CS	1	-	-	-	-	-
CSCA 1		500	1500	1500	-	-
CSCA 2		750	2250	2250	-	-
CSCA 3		1000	3000	3000	-	-
CSGTA 1		-	-	-	50	-
CSGTA 2		-	-	-	100	-
CSGTA 3		-	-	-	200	-
CSGP 1		-	-	-	-	25
CSGP 2		-	-	-	-	50
CSGP 3		-	-	-	-	100

*Note*: The numbers in the sample names are related to the number of reactants used for the crosslinking reaction.

**Table 2:** The results of the solubility and swelling tests under different pH conditions (CSCA 1/2/3 for CS:CA = 1:500/750/1000, CSGTA 1/2/3 for CS:GTA = 1:50/100/200, and CSGP 1/2/3 for CS:GP = 1:25/50/100 in their respective crosslinking reactions).

Sample	Solubility	Swelling Degree (%)
Neat CS	O	1013.5 ± 37.9
CSCA 1	O	424.1 ± 10.5
CSCA 2	X	501.1 ± 13.0
CSCA 3	X	593.4 ± 22.2
CSGTA 1	X	187.7 ± 2.3
CSGTA 2	X	168.2 ± 2.4
CSGTA 3	X	130.2 ± 7.1
CSGP 1	X	189.5 ± 5.5
CSGP 2	X	141.7 ± 4.2
CSGP 3	X	129.4 ± 5.4

**Table 3:** The UTM test results of the chitosan (CS) films using a 250 N load cell. The specimens were prepared according to ASTM D882.

Sample	Tensile Strength (MPa)	Elastic Modulus (MPa)	Elongation (%)
Neat CS	35.5 ± 1.1	34.5 ± 1.1	1.7 ± 0.2
CSCA 1	7.4 ± 0.9	5.7 ± 0.6	29.0 ± 2.8
CSCA 2	3.9 ± 0.4	2.7 ± 0.1	30.7 ± 1.7
CSCA 3	2.9 ± 0.1	2.2 ± 0.1	32.8 ± 1.8
CSGTA 1	82.7 ± 1.7	79.2 ± 1.2	4.4 ± 1.1
CSGTA 2	79.4 ± 1.1	77.1 ± 0.8	3.0 ± 0.4
CSGTA 3	72.1 ± 4.5	70.6 ± 4.3	2.0 ± 0.2
CSGP 1	74.2 ± 3.5	73.8 ± 2.9	4.9 ± 1.0
CSGP 2	71.8 ± 2.3	68.4 ± 1.5	4.8 ± 1.2
CSGP 3	74.1 ± 2.6	72.5 ± 2.4	2.1 ± 0.2

**Table 4:** Activated partial thromboplastin and whole blood clotting times of the crosslinked CS films and TB-CS films.

Sample	Activated Partial Thromboplastin Time (seconds, s)	Whole Blood Clotting Time (seconds, s)			
		Amounts of Coated Thrombin on the CS Films			
	0 µg	0 µg	320 µg	640 µg	960 µg
Control	70.3 ± 0.3	280.3 ± 20.3	-	-	-
Neat CS	69.0 ± 1.7	101.0 ± 15.3	-	-	-
CSCA 1	67.9 ± 3.9	91.0 ± 6.2	44.0 ± 6.0	27.5 ± 0.4	27.0 ± 1.4
CSCA 2	63.9 ± 3.8	89.3 ± 5.5	35.0 ± 5.6	26.5 ± 2.1	26.0 ± 1.8
CSCA 3	67.3 ± 2.1	89.7 ± 5.4	38.5 ± 4.6	28.5 ± 0.4	25.0 ± 0.7
CSGTA 1	66.0 ± 1.8	91.0 ± 6.4	45.0 ± 1.8	20.0 ± 3.5	19.0 ± 4.2
CSGTA 2	66.3 ± 1.1	94.0 ± 4.2	41.5 ± 0.4	20.0 ± 2.8	19.0 ± 0.7
CSGTA 3	68.2 ± 0.5	81.5 ± 3.9	44.0 ± 6.0	16.5 ± 1.1	16.5 ± 0.4
CSGP 1	63.6 ± 0.7	99.5 ± 1.1	47.5 ± 2.5	18.5 ± 0.3	16.0 ± 0.7
CSGP 2	64.8 ± 3.9	91.0 ± 6.4	47.0 ± 1.4	19.5 ± 0.4	15.0 ± 0.7
CSGP 3	63.6 ± 1.7	91.3 ± 8.0	46.0 ± 3.2	15.5 ± 1.2	16.5 ± 0.4
